# Supplementary material for: Developing and validating COVID-19 adverse outcome risk prediction models from a bi-national European cohort of 5594 patients
Source: Sci Rep. 2021 Feb 5;11:3246. doi: 10.1038/s41598-021-81844-x (PMC7864944; doi:10.1038/s41598-021-81844-x)
Supplement: Supplementary file 1 — Supplementary Information 1. [file 41598_2021_81844_MOESM1_ESM.docx]

**DEVELOPING AND VALIDATING COVID-19 ADVERSE OUTCOME RISK PREDICTION MODELS FROM A BI-NATIONAL EUROPEAN COHORT OF 5594 PATIENTS.**

Espen Jimenez-Solem MD PhD^1,13^, Tonny S Petersen MD PhD^1,13^, Casper Hansen MSc PhD ^2^, Christian Hansen MSc, PhD ^2^,Christina Lioma MSc PhD^2^, Christian Igel MSc PhD ^2^, Wouter Boomsma MSc PhD ^2^, Oswin Krause MSc PhD ^2^, Stephan Lorenzen MSc PhD ^2^, Raghavendra Selvan MSc PhD ^2^, Janne Petersen MD PhD^11,12^, Martin Erik Nyeland Msc PhD^1^, Mikkel Zöllner Ankarfeldt PhD^11^, Gert Mehl Virenfeldt MSc^11^, Matilde Winther-Jensen MSc PhD^11^, Allan Linneberg MD PhD^11^, Mostafa Mediphour Ghazi MSc PhD ^2^, Nicki Detlefsen MSc PhD ^2,3^, Andreas Lauritzen MSc PhD ^2^, Abraham George Smith MSc PhD ^2^, Marleen de Bruijne MSc PhD ^2,14^, Bulat Ibragimov MSc PhD ^2^, Jens Petersen MSc PhD ^2^, Martin Lillholm MSc PhD ^2^, Jon Middleton MSc PhD ^2^, Stine Hasling Mogensen MSc PhD ^4^, Hans-Christian Thorsen-Meyer MD^5^, Anders Perner MD PhD ^5^, Marie Helleberg MD PhD DMSc ^6^, Benjamin Skov Kaas-Hansen MD^7^, Mikkel Bonde^10^, Alexander Bonde MD^9,10^, Akshay Pai MSc PhD ^2,8,^, Mads Nielsen MSc PhD ^2^ and Martin Sillesen MD PhD ^9,10,13^

1. Department of Clinical Pharmacology, Copenhagen University Hospital, Bispebjerg and Frederiksberg, Denmark
2. Department of Computer Science, University of Copenhagen, Denmark
3. DTU Compute. Denmarks Technical University, Lyngby, Denmark
4. Danish Medicines Agency, Copenhagen Denmark
5. Department of Intensive Care Medicine, Copenhagen University Hospital, Rigshospitalet, Denmark
6. Department of Infectious Diseases, Copenhagen University Hospital, Rigshospitalet, Denmark
7. Clinical Pharmacology Unit, Zealand University Hospital, Roskilde Denmark
8. Cerebriu A/S. Copenhagen, Denmark
9. Department of Surgical Gastroenterology, Copenhagen University Hospital, Rigshospitalet, Denmark
10. Center for Surgical Translational and Artificial Intelligence Research (CSTAR), Copenhagen University Hospital, Rigshospitalet, Denmark.
11. Center for Clinical Research and Prevention, Copenhagen University Hospital, Bispebjerg and Frederiksberg, Denmark
12. Section of Biostatistics, Department of Public Health, University of Copenhagen, Denmark
13. Department of Clinical Medicine, University of Copenhagen, Denmark
14. Department of Radiology and Nuclear Medicine, Erasmus MC – University Medical Center Rotterdam, The Netherlands.

**SUPPLEMENTARY TABLES**

**Supplementary Table S1:** International Classification of Diseases (ICD) 10 and Anatomical Therapeutic Classification (ATC) codes used of the definition of chosen comorbidities. ICD-10 codes list the presence of a given comorbidity in the Electronic Health Record, whereas the ATC code indicates that the patient is on a drug targeting the specific comorbidity.
A comorbidity was classified if either the ICD-10 or ATC code was present for the given patient.

COPD: Chronic Obstructive Lung Disease

Neurological manifestations: Pre-existing neurodegenerative diseases such as Parkinson’s disease.

| Comorbidity | Included ICD-10 codes | Excluded ICD-10 codes | Included ATC codes | Excluded ATC codes |
| --- | --- | --- | --- | --- |
| Diabetes | E10,E11,E12,E13,E14,H28.0  H36.0 |  | A10 |  |
| Ischemic Heart Disease | I20, I212, I23, I24, I25 |  | CO1D |  |
| Arrythmias | I47, I48, I49 |  | CO1AA05 |  |
| Heart Failure | I50. I42.0, I42.2, I42.6, I42.7, I42.8, I42.9, I11.0, I13.0, I13.2, |  | CO9DX04 |  |
| Stroke | G45, G46, I60, I62, I63, I64, I65, I66, I67, I68, I69 |  | BO1AC30, BO1AC07 |  |
| COPD or Asthma | J40, J41, J42, J43, J44, J45, J46, J47 |  |  |  |
| Sleep apnea | G47.3 |  |  |  |
| Arthritis | MO5, MO.6 |  |  |  |
| Osteoporosis | M80, M81, M82 |  | MO5B, GO3XC01, HO5AA02, HO5AA03 |  |
| Dementia | F00, G30, F01, F02.0, F03.9, G31.8B, G31.8E, G31.9, G31.0B |  | NO6D |  |
| Mental disorder | F20, F21, F22, F25, F28, F29, F31 |  |  |  |
| Immunodeficiency | D80, D81, D82, D83, D84 |  |  |  |
| Neurological Manifestations | G | G45, G46 |  |  |
| Cancer |  |  |  | LO1AA01,  LO1BA01,  LO1X02 |
| Chronic Kidney Failure | N18 | N181 |  |  |

Supplementary Table S2: List of extracted laboratory tests, where available.

| **Group** | **Test** | **Abbreviation** | **Analysed from** |
| --- | --- | --- | --- |
| Inflammation/Infection | C-reactive protein | CRP | Plasma |
|  | Lymphocyte Count | - | Blood |
|  | Leucocyte Count | - | Blood |
|  | Neutrophil Count | - | Blood |
| Liver | Lactate Dehydrogenase | LDH | Plasma |
|  | Alanine Aminotransferase | ALAT | Plasma |
| Coagulation and blood | D-Dimer | - | Plasma |
|  | Hemoglobin | Hgb | Blood |
|  | Ferritine | - | Plasma |
| Renal | Blood Urea Nitrogen | BUN | Plasma |
|  | Creatinine | - | Plasma |
| Arterial Blood gas | Base Excess | BE | Plasma |
|  | Bicarbonate | - | Plasma |
|  | Lactate | - | Plasma |
|  | pO2 | - | Plasma |
|  | pCO2 | - | Plasma |
|  | pH | - | Plasma |

Supplementary Table S3: List of extracted vital signs

| Pulse |
| --- |
| Temperature |
| Diastolic Blood Pressure |
| Systolic blood pressure |
| Early Warning Score |
| Oxygen supplementation (liters per minute) |
| Oxygen Saturation |

Supplementary Table S4: Missing data in percentages

|  | All patients | Non-hospitalized | Hospitalized patients | Hospitalized patients without ICU admission | Hospitalized patients with ICU admission | Survivors | Non-survivors |
| --- | --- | --- | --- | --- | --- | --- | --- |
| CRP (mean) | 68.9 | 98.2 | 13.1 | 13.3 | 11.6 | 73.6 | 15.4 |
| CRP (slope) | 91.1 | 99.8 | 74.6 | 81 | 33.1 | 92.8 | 71.9 |
| CRP (most recent) | 68.9 | 98.2 | 13.1 | 13.3 | 11.6 | 73.6 | 15.4 |
| Lymphocyte Count (mean) | 71.1 | 98.3 | 19.3 | 19.5 | 17.7 | 75.5 | 22.2 |
| Lymphocyte Count (slope) | 92.8 | 100 | 79.3 | 85.3 | 40.3 | 94.2 | 77.2 |
| Lymphocyte Count (most recent) | 71.1 | 98.3 | 19.4 | 19.6 | 17.7 | 75.5 | 22.2 |
| Lactic dehydrogenase (mean) | 74.3 | 98.8 | 27.7 | 28.8 | 21 | 78.1 | 31.2 |
| Lactic dehydrogenase (slope) | 95.6 | 100 | 87.4 | 92.6 | 53.6 | 96.4 | 87.3 |
| Lactic dehydrogenase (most recent) | 74.3 | 98.8 | 27.7 | 28.8 | 21 | 78.1 | 31.2 |
| Alanine aminotransferase (mean) | 71.2 | 98.5 | 19.2 | 19.9 | 14.9 | 75.6 | 21.9 |
| Alanine aminotransferase (slope) | 94.5 | 100 | 84.2 | 89.8 | 47.5 | 95.6 | 82.4 |
| Alanine aminotransferase (most recent) | 71.2 | 98.5 | 19.2 | 19.9 | 14.9 | 75.6 | 21.9 |
| Hemoglobin (mean) | 68.5 | 97.8 | 12.7 | 13 | 11 | 73.2 | 15.4 |
| Hemoglobin (slope) | 80 | 99.3 | 43.2 | 46.9 | 19.3 | 84.1 | 33.6 |
| Hemoglobin (most recent) | 68.5 | 97.8 | 12.7 | 13 | 11 | 73.2 | 15.4 |
| White blood cells (mean) | 69 | 98.1 | 13.6 | 13.9 | 11.6 | 73.8 | 16 |
| White blood cells (slope) | 91.2 | 100 | 74.6 | 81.2 | 32 | 93 | 70.1 |
| White blood cells (most recent) | 69.3 | 98.1 | 14.6 | 14.6 | 14.4 | 74 | 17 |
| Neutrophil (mean) | 71 | 98.3 | 19.1 | 19.4 | 17.7 | 75.4 | 21.9 |
| Neutrophil (slope) | 92.4 | 100 | 78.3 | 84.8 | 35.9 | 94 | 75.3 |
| Neutrophil (most recent) | 71 | 98.3 | 19.1 | 19.4 | 17.7 | 75.4 | 21.9 |
| D dimer (mean) | 92.1 | 99.8 | 77.5 | 80.3 | 59.1 | 93.4 | 78.1 |
| D dimer (slope) | 98.9 | 100 | 97 | 99.3 | 81.8 | 99.2 | 96 |
| D dimer (most recent) | 92.1 | 99.8 | 77.5 | 80.3 | 59.1 | 93.4 | 78.1 |
| Blood urea nitrogen (mean) | 72.5 | 98.8 | 22.4 | 23.1 | 17.7 | 76.8 | 24.4 |
| Blood urea nitrogen (slope) | 94.9 | 100 | 85.4 | 91 | 48.6 | 95.9 | 83.6 |
| Blood urea nitrogen (most recent) | 72.5 | 98.8 | 22.4 | 23.1 | 17.7 | 76.8 | 24.4 |
| Creatinine (mean) | 69 | 98.3 | 13.2 | 13.5 | 11.6 | 73.8 | 15.1 |
| Creatinine (slope) | 89.6 | 100 | 70.1 | 76.1 | 30.9 | 92.1 | 62 |
| Creatinine (most recent) | 69 | 98.3 | 13.2 | 13.5 | 11.6 | 73.8 | 15.1 |
| Ferritin (mean) | 90.7 | 99.7 | 73.6 | 76.1 | 57.5 | 92.1 | 74.4 |
| Ferritin (slope) | 98.4 | 100 | 95.5 | 98.2 | 77.9 | 98.8 | 94.8 |
| Ferritin (most recent) | 90.7 | 99.7 | 73.6 | 76.1 | 57.5 | 92.1 | 74.4 |
| Base excess (mean) | 80.9 | 99.1 | 46.4 | 50.5 | 19.3 | 84.8 | 38 |
| Base excess (slope) | 92.2 | 99.7 | 77.9 | 85 | 32 | 94.4 | 68.2 |
| Base excess (most recent) | 81 | 99.1 | 46.6 | 50.5 | 21 | 84.9 | 38 |
| HCO₃ (mean) | 80.4 | 99 | 44.9 | 49 | 18.2 | 84.4 | 35.8 |
| HCO₃ (slope) | 92.3 | 99.8 | 78.1 | 85.1 | 32 | 94.4 | 68.5 |
| HCO₃ (most recent) | 80.4 | 99 | 44.9 | 49 | 18.2 | 84.4 | 35.8 |
| Lactate (mean) | 85.3 | 99.1 | 59 | 62.4 | 37 | 88.2 | 52.8 |
| Lactate (slope) | 93.9 | 99.7 | 82.8 | 88.3 | 47 | 95.6 | 75.3 |
| Lactate (most recent) | 85.3 | 99.1 | 59 | 62.4 | 37 | 88.2 | 52.8 |
| O₂ (mean) | 81.8 | 99.1 | 48.8 | 53.2 | 19.9 | 85.6 | 38.9 |
| O₂ (slope) | 92.8 | 99.8 | 79.6 | 86.5 | 34.8 | 94.8 | 71.6 |
| O₂ (most recent) | 81.8 | 99.1 | 48.8 | 53.2 | 19.9 | 85.6 | 38.9 |
| pCO₂ (mean) | 81.8 | 99.1 | 48.7 | 53.1 | 19.9 | 85.6 | 38.9 |
| pCO₂ (slope) | 92.7 | 99.8 | 79.4 | 86.4 | 33.7 | 94.7 | 70.7 |
| pCO₂ (most recent) | 81.8 | 99.1 | 48.7 | 53.1 | 19.9 | 85.6 | 38.9 |
| ph (mean) | 81.8 | 99.1 | 48.7 | 53.1 | 19.9 | 85.6 | 38.9 |
| ph (slope) | 93.1 | 99.8 | 80.2 | 86.9 | 36.5 | 94.9 | 71.9 |
| ph (most recent) | 81.8 | 99.1 | 48.7 | 53.1 | 19.9 | 85.6 | 38.9 |
| pO₂ (mean) | 81.8 | 99.1 | 48.8 | 53.2 | 19.9 | 85.6 | 38.9 |
| pO₂ (slope) | 92.4 | 99.8 | 78.5 | 85.6 | 32.6 | 94.5 | 69.4 |
| pO₂ (most recent) | 81.8 | 99.1 | 48.8 | 53.2 | 19.9 | 85.6 | 38.9 |
| Pulse (mean) | 66.5 | 95.2 | 12 | 12.1 | 11 | 71.2 | 14.8 |
| Pulse (slope) | 72.2 | 99.3 | 20.6 | 21.6 | 13.8 | 76.8 | 20.4 |
| Pulse (most recent) | 66.5 | 95.2 | 12 | 12.1 | 11 | 71.2 | 14.8 |
| Temperature (mean) | 66.7 | 95.5 | 11.8 | 12 | 11 | 71.3 | 15.1 |
| Temperature (slope) | 72.8 | 99.5 | 22 | 23 | 15.5 | 77.2 | 24.1 |
| Temperature (most recent) | 66.7 | 95.5 | 11.8 | 12 | 11 | 71.3 | 15.1 |
| Early Warning Score (mean) | 70.9 | 97.9 | 19.6 | 20.6 | 13.3 | 75.7 | 17.9 |
| Early Warning Score (slope) | 76.1 | 99.7 | 31.3 | 33.5 | 17.1 | 80.5 | 27.5 |
| Early Warning Score (most recent) | 74 | 98 | 28.3 | 30.4 | 14.9 | 78.6 | 22.8 |
| Respiratory rate (mean) | 66.9 | 95.6 | 12.5 | 12.6 | 11.6 | 71.5 | 15.4 |
| Respiratory rate (slope) | 75 | 99.6 | 28.3 | 30.2 | 16 | 79.4 | 26.2 |
| Respiratory rate (most recent) | 66.9 | 95.6 | 12.5 | 12.6 | 11.6 | 71.5 | 15.4 |
| Saturation (mean) | 66.5 | 95.2 | 11.8 | 12 | 11 | 71.1 | 14.8 |
| Saturation (slope) | 74.4 | 99.3 | 27.1 | 28.9 | 15.5 | 78.8 | 25.6 |
| Saturation (most recent) | 66.5 | 95.2 | 11.8 | 12 | 11 | 71.1 | 14.8 |

**Supplementary Table S5:** Demographic information on the group SARS-CoV-2 positive patients from the United Kingdom biobank cohort used for external validation. The table presents information on the full cohort (admitted and non-admitted SARS-CoV-2 positive patients) as well as subgroups admitted to a hospital and Intensive Care Unit (ICU) respectively. Furthermore, differential demographics between survivors and non-survivors (in-hospital mortality) is presented. Continuous variables are presented as medians with (interquartile range)
**p<0.001 when subgroups are compared (e.g. hospitalized vs. non-hospitalized, ICU vs. non-ICU, survivors vs. non- survivors).

COPD: Chronic Obstructive Pulmonary Disease.

|  | All SARS-CoV-2  patients  (n=1650) | Non-hospitalized (n=897) | Hospitalized patients  (n=753) | Hospitalized patients without ICU admission  (n=1519) | Hospitalized patients  with ICU admission  (n=131) | Survivors  (n=1345) | Non-survivors  (n=305) |
| --- | --- | --- | --- | --- | --- | --- | --- |
| Body Mass  Index | 28.0 (25.1-31.5) | 27.3 (24.6-30.5) | 28.8 (25.9-32.6)** | 27.8 (25.0-31.2) | 29.9 (27.1-33.8)** | 27.7 (25.0-31.2) | 28.8 (25.9-32.6) |
| Age | 69.0 (59.0-76.0) | 64.0 (57.0-75.0) | 73.0 (67.0-78.0)** | 69.0 (59.0-77.0) | 69.0 (61.5-75.0) | 66.0 (58.0-75.0) | 76.0 (71.0-79.0)** |
| Male Sex | 47.8 | 56.6 | 37.2** | 49.0 | 32.8** | 50.7 | 34.8** |
| Diabetes | 14.1 | 10.3 | 18.7** | 14.3 | 12.2 | 12.1 | 23.0** |
| Ischemic heart disease | 14.7 | 10.6 | 19.7** | 14.8 | 13.7 | 12.6 | 24.3** |
| Heart failure | 4.2 | 2.7 | 6.0** | 4.2 | 3.8 | 3.4 | 7.5** |
| Arrhythmia | 9.9 | 6.8 | 13.5** | 10.0 | 8.4 | 8.4 | 16.4** |
| Stroke | 5.9 | 5.8 | 6.1 | 6.3 | 1.5 | 5.0 | 10.2** |
| COPD or Asthma | 15.2 | 12.2 | 18.7** | 15.1 | 15.3 | 13.8 | 21.0** |
| Sleep apnoea | 0.0 | 0.0 | 0.0 | 0.0 | 0.0 | 0.0 | 0.0 |
| Arthritis | 2.4 | 1.6 | 3.5 | 2.4 | 3.1 | 1.8 | 5.2** |
| Osteoporosis | 3.0 | 2.5 | 3.7 | 3.1 | 2.3 | 2.8 | 3.9 |
| Dementia | 2.1 | 2.5 | 1.7 | 2.3 | 0.0 | 1.6 | 4.3 |
| Severe mental disorder | 0.8 | 0.7 | 1.1 | 0.8 | 1.5 | 0.6 | 2.0 |
| Immunodeficiencies | 0.3 | 0.3 | 0.3 | 0.3 | 0.0 | 0.2 | 0.7 |
| Neurological manifestations | 19.2 | 16.6 | 22.3 | 19.6 | 14.5 | 17.2 | 27.9** |
| Cancer | 12.8 | 10.3 | 15.9** | 12.9 | 12.2 | 11.4 | 19.3** |
| Chronic kidney failure | 5.2 | 3.7 | 7.0 | 5.1 | 6.1 | 4.6 | 7.9 |
| Dialysis | 0.9 | 1.1 | 0.7 | 0.9 | 0.8 | 1.0 | 0.7 |
| Hypertension | 35.5 | 28.2 | 44.1** | 35.3 | 37.4 | 31.1 | 54.8** |

**Supplementary Table S6:** Main results from the external validation of the prediction models.

Performance metrics are presented as the Receiver Operating Characteristics Area Under the Curve (ROC-AUC) for True/False positive rates (TPR/FPR) and Precision/Recall (Pre/Rec).

*Model is significantly (p<0.01) better than the base prediction model (Age+gender+Body Mass Index, BMI)

--: Insufficient data available at the time point, or prediction irrelevant (e.g. predicting hospital admission for patients already in the ICU).

|  | Hospital admission |  | ICU admission |  | Death |  |
| --- | --- | --- | --- | --- | --- | --- |
|  | TPR/FPR | Pre/Rec | TPR/FPR | Pre/Rec | TPR/FPR | Pre/Rec |
| Diagnoses |  |  |  |  |  |  |
| Age+Gender+BMI | 0.648 | 0.560 | 0.501 | 0.087 | 0.715 | 0.293 |
| +Comorbidities | 0.661 | 0.579 | 0.529 | 0.088 | 0.742* | 0.328 |
| Admission |  |  |  |  |  |  |
| Age+Gender+BMI | -- | -- | 0.565 | 0.196 | 0.621 | 0.400 |
| +Comorbidities | -- | -- | 0.518 | 0.192 | 0.617 | 0.410 |
| Pre-ICU |  |  |  |  |  |  |
| Age+Gender+BMI | -- | -- | -- | -- | 0.617 | 0.525 |
| +Comorbidities | -- | -- | -- | -- | 0.722* | 0.655 |
| Post-ICU |  |  |  |  |  |  |
| Age+Gender+BMI | -- | -- | -- | -- | 0.617 | 0.525 |
| +Comorbidities | -- | -- | -- | -- | 0.705 | 0.639 |

**SUPPLEMENTARY FIGURES**

Supplementary Figure S1: Receiver operator characteristics – area under the curve (ROC-AUC) of the patients tested positive for SARS-CoV-2 (Diagnosis model). The model predicted risks of hospital admission (bottom left), intensive care admission (bottom right), ventilator treatment (top right) and death (top left). Orange line indicates results obtained on Danish data; green line indicates results obtained in external validation data from the UK biobank.

**Supplementary Figure S2:** Receiver operator characteristics – area under the curve (ROC-AUC) of the patients admitted to the hospital. Model inputs included data available up to 12 hours after the admission timepoint (admission model). The model predicted risks of intensive care admission (bottom), ventilator treatment (top right) and death (top left).

Orange line indicates results obtained on Danish data; green line indicates results obtained in external validation data from the UK biobank.

Supplementary Figure S3: Receiver operator characteristics – area under the curve (ROC-AUC) of the patients admitted to the Intensive Care Unit (ICU). Model inputs included data available from the admission model, as well as temporal features available during the 12 hours leading up to ICU admission. The model predicted risks of ventilator treatment (right) and death (left).

Orange line indicates results obtained on Danish data; green line indicates results obtained in external validation data from the UK biobank.

Supplementary Figure S4: Receiver operator characteristics – area under the curve (ROC-AUC) of the patients admitted to the Intensive Care Unit (ICU). Model inputs included data available from the admission model, as well as temporal features available during the 12 hours after ICU admission. The model predicted risks of ventilator treatment (right) and death (left).

Orange line indicates results obtained on Danish data; green line indicates results obtained in external validation data from the UK biobank.

Supplementary Figure S5: Feature importance for the basic (including age, sex and body mass index) and advanced (all data) pre-ICU model, predicting risk ventilator treatment (first row) and death (second row).

Supplementary Figure S6: Feature importance for the basic (including age, sex and body mass index) and advanced (all data) post-ICU model, predicting risk ventilator treatment (first row) and death (second row).

**Supplementary Figure S7:** Calibration curves for basic models. Orange line indicates Danish data, green line indicates data from the United Kingdom biobank. For each calibration plot, the abscissa shows the mean of the predictions within a bin and the ordinate shows the fraction of positive labels for the corresponding samples.

**Supplementary Figure S8:** Calibration curves for advanced models. Orange line indicates Danish data. For each calibration plot, the abscissa shows the mean of the predictions within a bin and the ordinate shows the fraction of positive labels for the corresponding samples.
