# Supplementary figures and images for: Developing and validating COVID-19 adverse outcome risk prediction models from a bi-national European cohort of 5594 patients
Source: Sci Rep. 2021 Feb 5;11:3246. doi: 10.1038/s41598-021-81844-x (PMC7864944; doi:10.1038/s41598-021-81844-x)

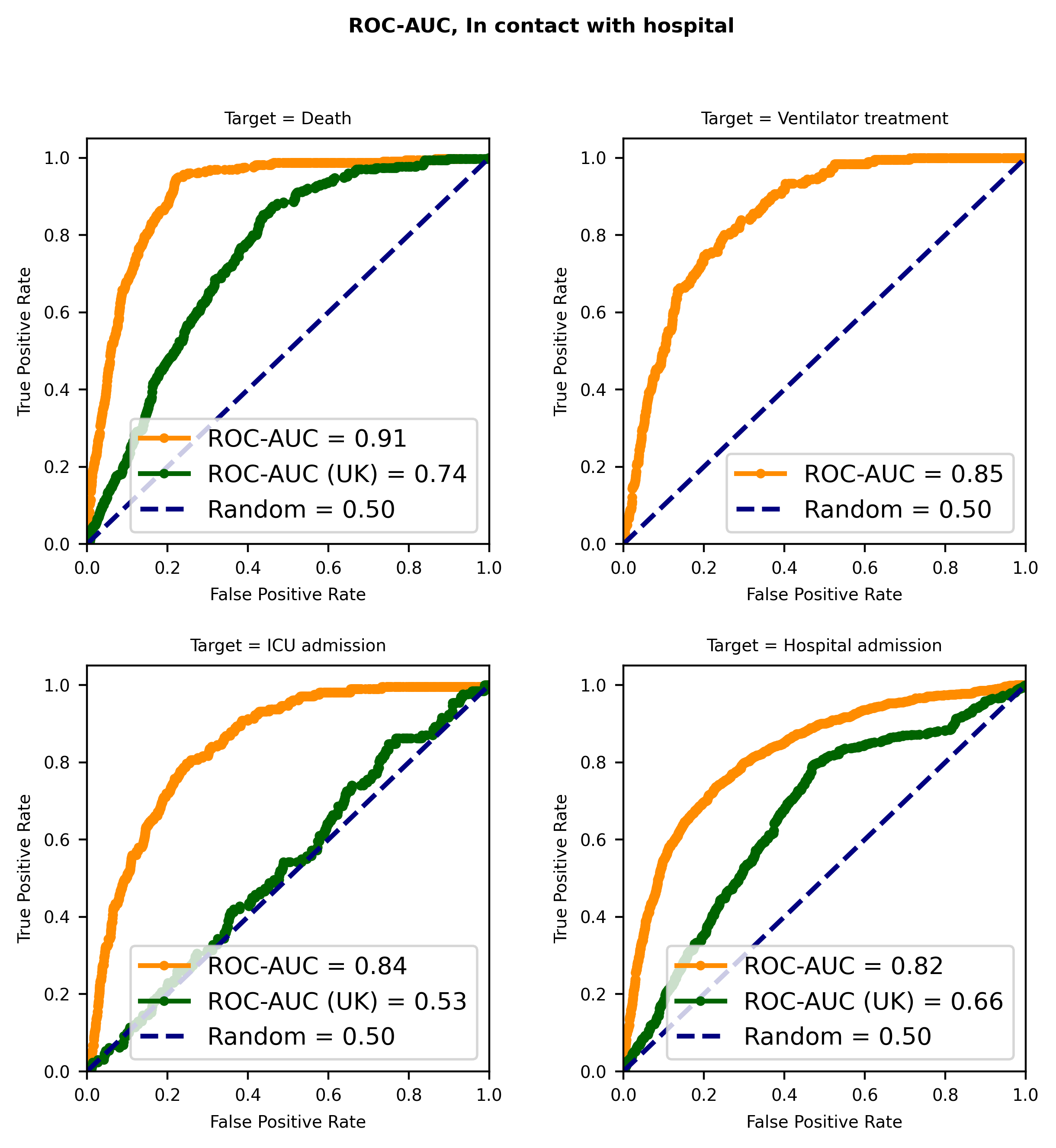

Supplement: Supplementary file 2 — Supplementary Figure S1. [file 41598_2021_81844_MOESM2_ESM.png]

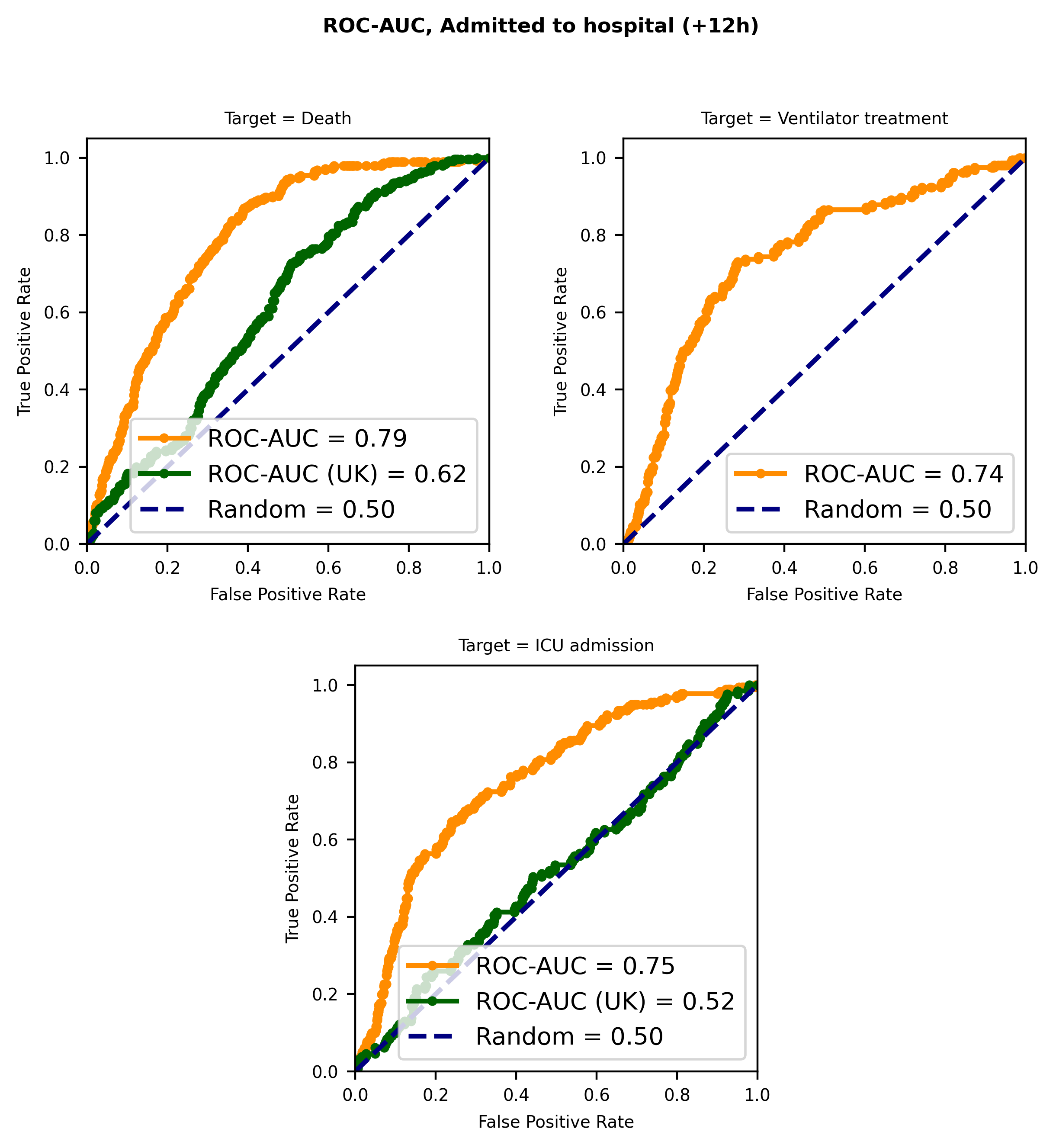

Supplement: Supplementary file 3 — Supplementary Figure S2. [file 41598_2021_81844_MOESM3_ESM.png]

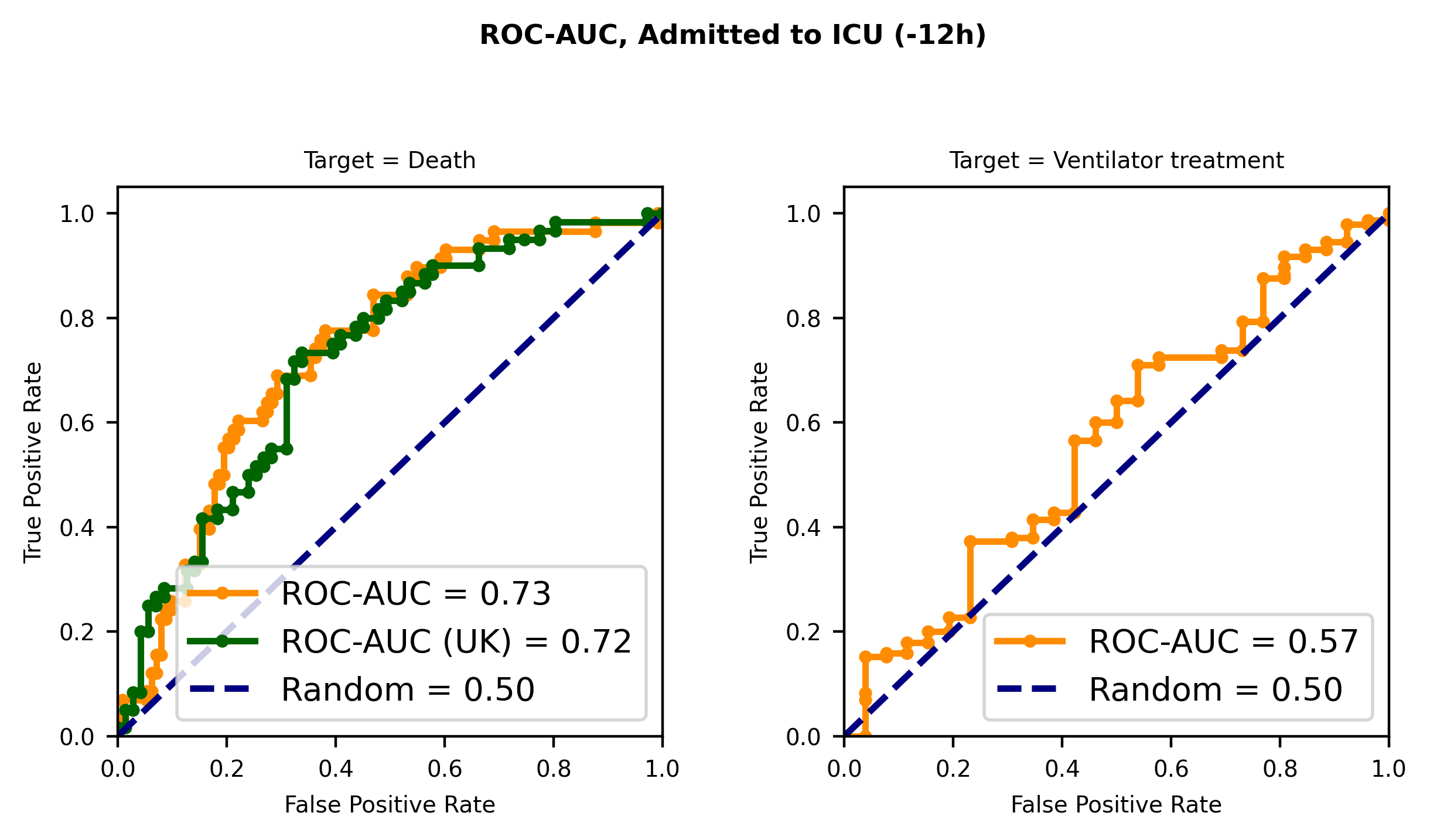

Supplement: Supplementary file 4 — Supplementary Figure S3. [file 41598_2021_81844_MOESM4_ESM.png]

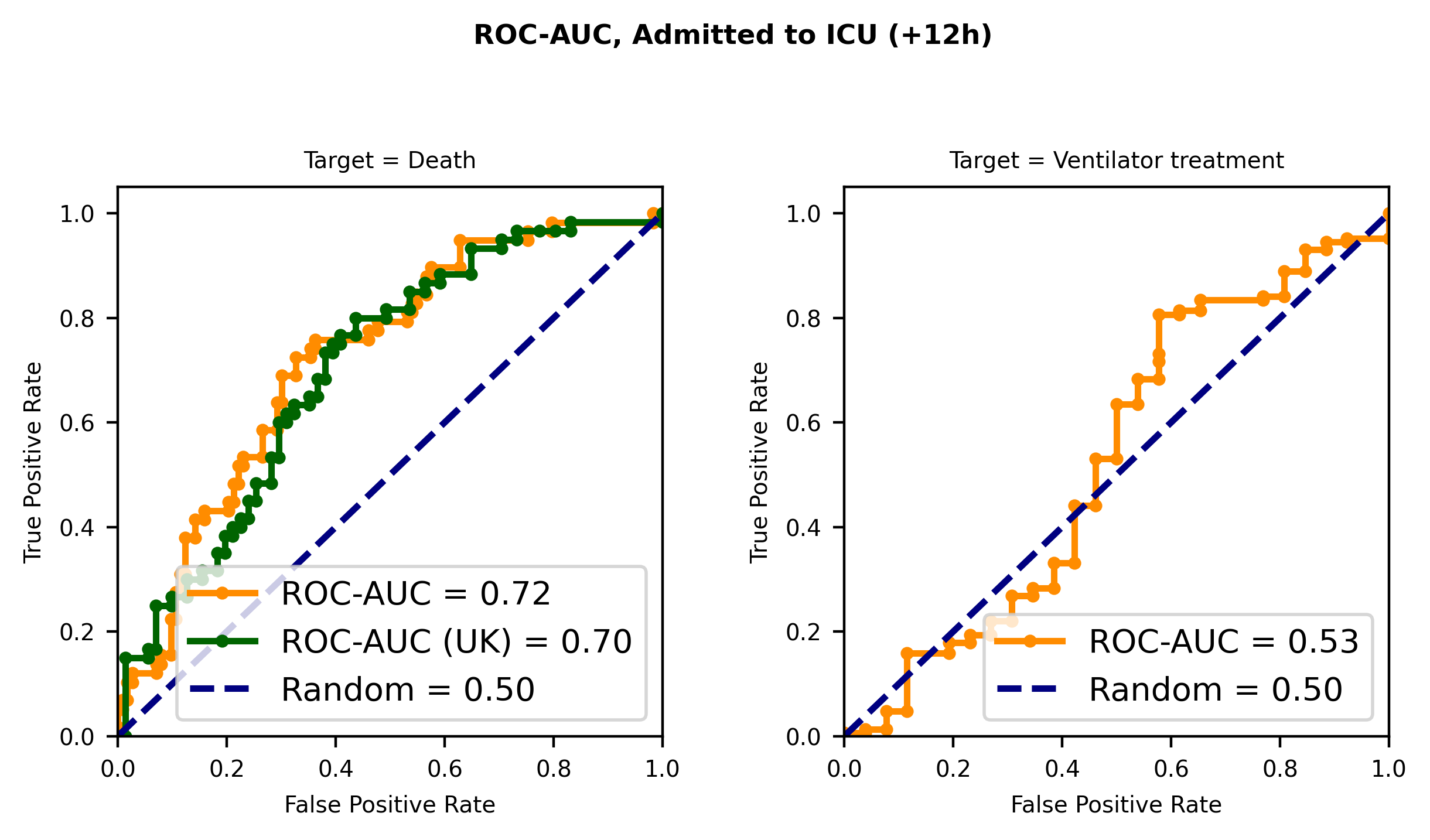

Supplement: Supplementary file 5 — Supplementary Figure S4. [file 41598_2021_81844_MOESM5_ESM.png]

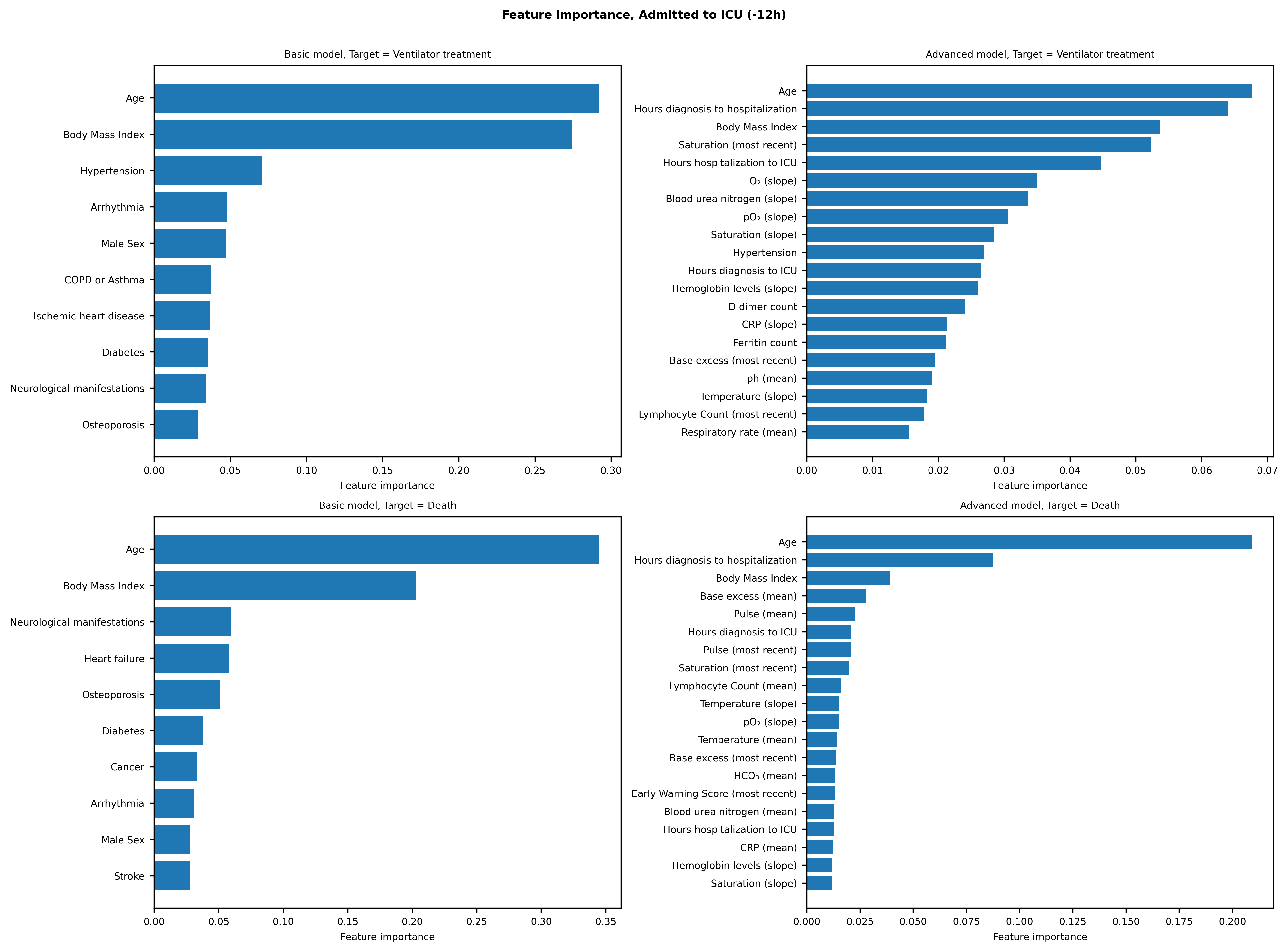

Supplement: Supplementary file 6 — Supplementary Figure S5. [file 41598_2021_81844_MOESM6_ESM.png]

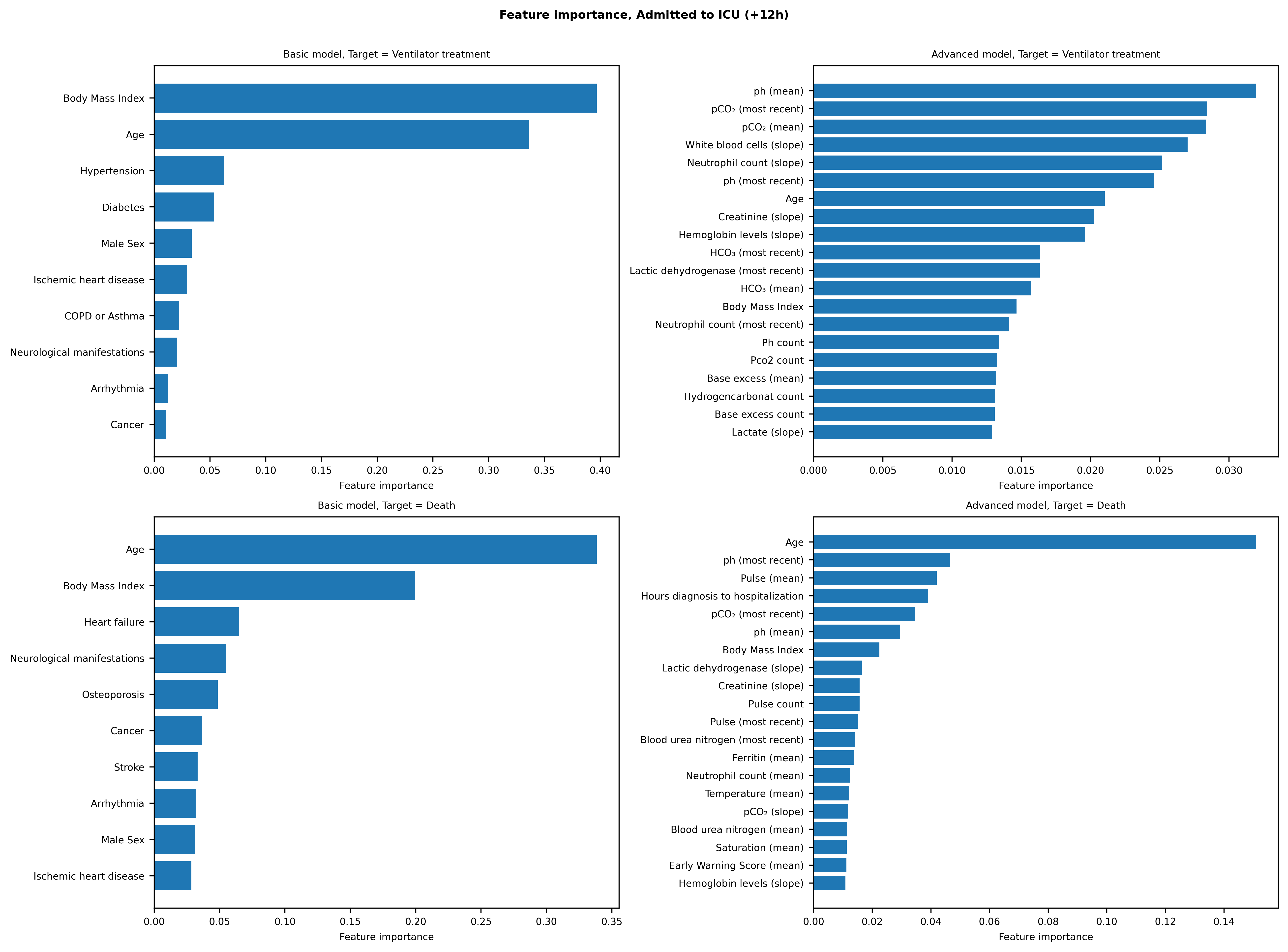

Supplement: Supplementary file 7 — Supplementary Figure S6. [file 41598_2021_81844_MOESM7_ESM.png]

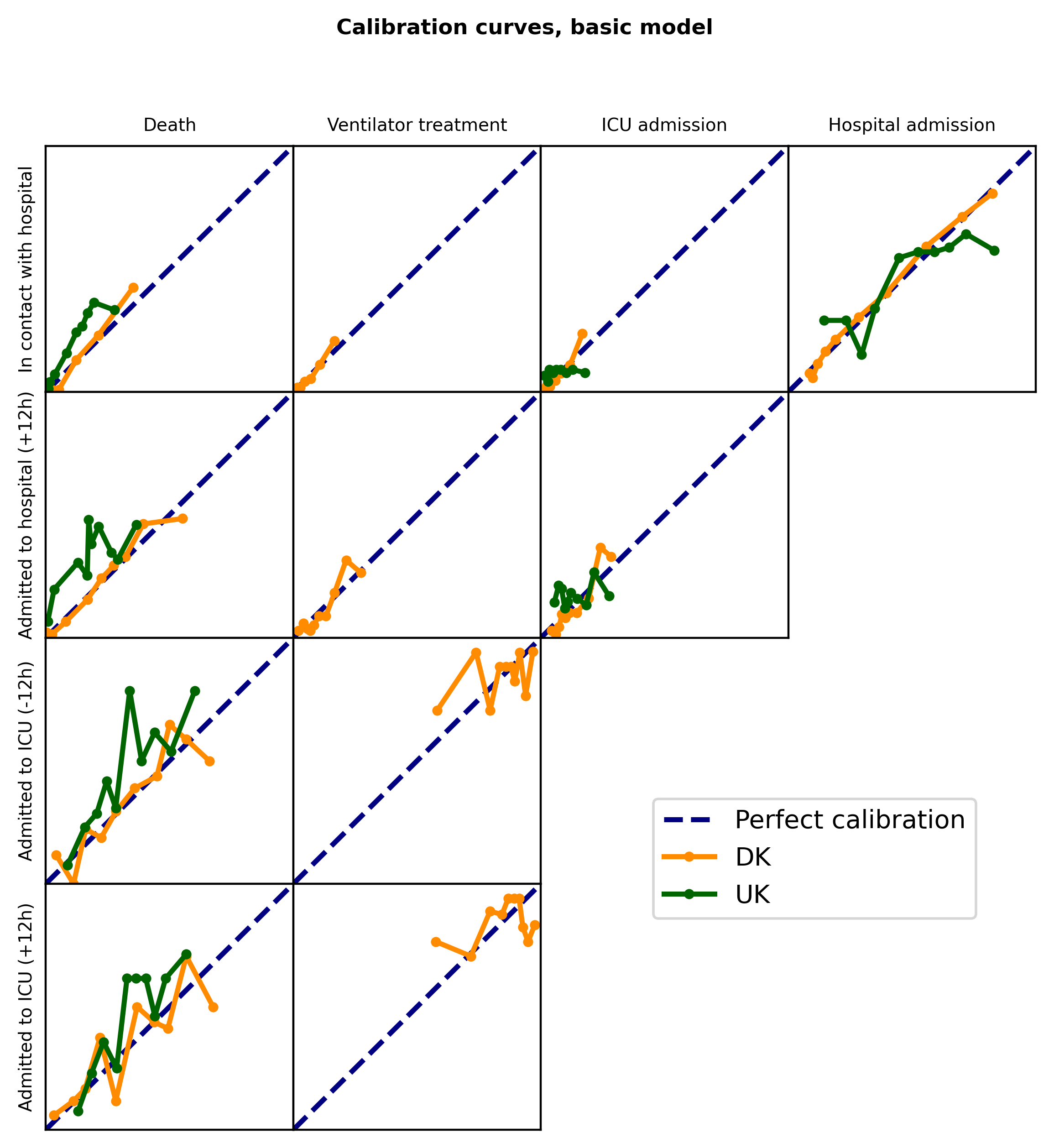

Supplement: Supplementary file 8 — Supplementary Figure S7. [file 41598_2021_81844_MOESM8_ESM.png]

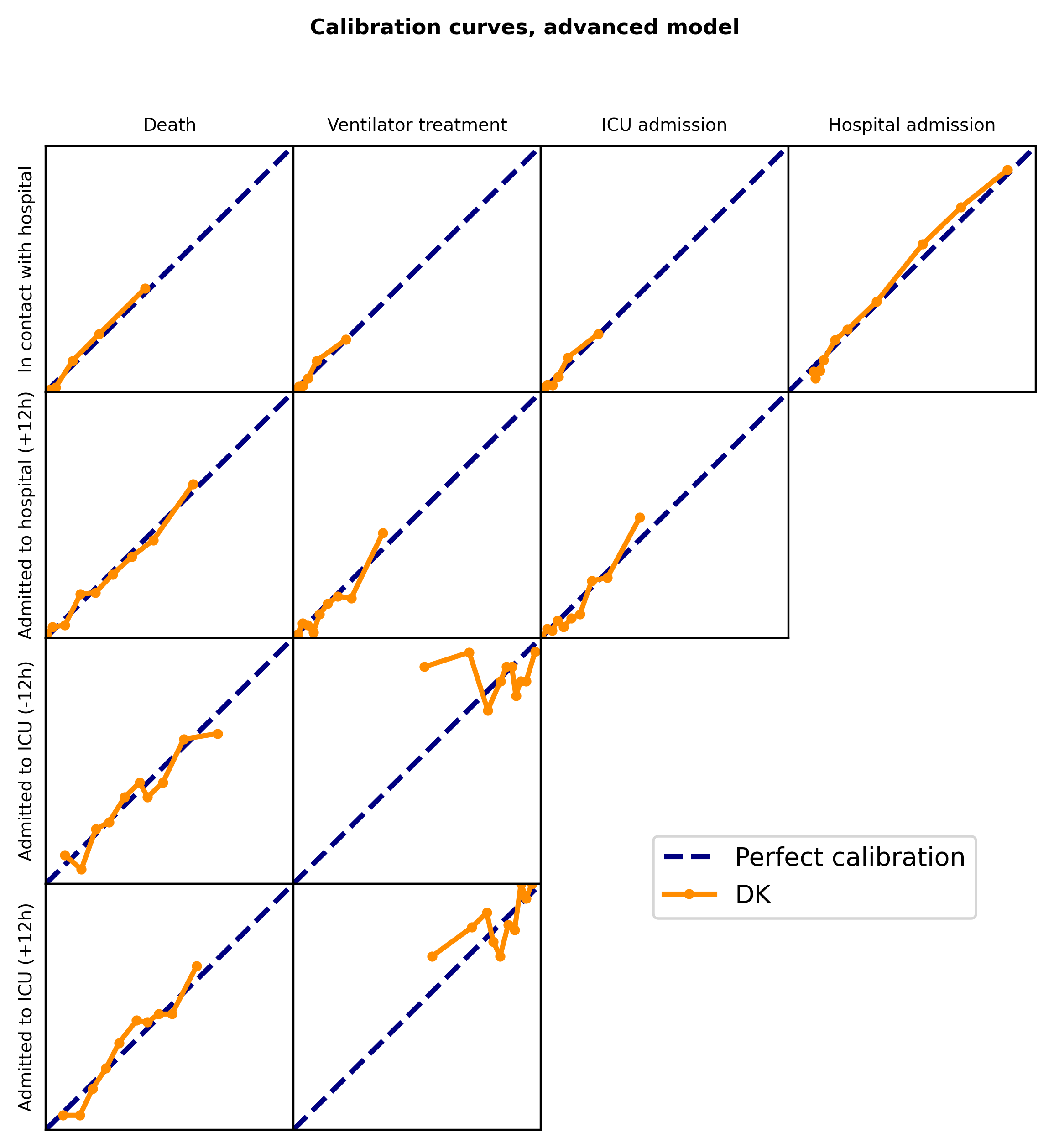

Supplement: Supplementary file 9 — Supplementary Figure S8. [file 41598_2021_81844_MOESM9_ESM.png]
